# Anti-Cancer and Anti-Inflammatory Potential of the Green Synthesized Silver Nanoparticles of the Red Sea Sponge *Phyllospongia lamellosa* Supported by Metabolomics Analysis and Docking Study

**DOI:** 10.3390/antibiotics10101155

**Published:** 2021-09-24

**Authors:** Areej A. Al-Khalaf, Hossam M. Hassan, Aisha M Alrajhi, Rania Ali El Hadi Mohamed, Wael N. Hozzein

**Affiliations:** 1 Department of Biology, College of Science, Princess Nourah Bint Abdulrahman University, Riyadh 11671, Saudi Arabia; amo.alrajhi@gmail.com (A.M.A.); r.ali2012@yahoo.com (R.A.E.H.M.); 2 Department of Pharmacognosy, Faculty of Pharmacy, Nahda University, Beni-Suef 62513, Egypt; 3 Department of Pharmacognosy, Faculty of Pharmacy, Beni-Suef University, Beni-Suef 62514, Egypt; 4 Bioproducts Research Chair, Zoology Department, College of Science, King Saud University, Riyadh 11671, Saudi Arabia; hozzein29@yahoo.com; 5 Botany and Microbiology Department, Faculty of Science, Beni-Suef University, Beni-Suef 62514, Egypt

**Keywords:** *Phyllospongia lamellosa*, silver nanoparticles, metabolomics, anti-cancer, antibacterial, anti-inflammatory

## Abstract

Background: The Red Sea sponges have been endorsed as a plentiful source of bioactive compounds with promising anti-cancer and anti-inflammatory activities; therefore, exploring their potential as a source of anti-cancer metabolites has stimulated a growing research interest. Purpose: To investigate the anti-cancer and anti-inflammatory potential of the Red Sea sponges, in their bulk and silver nanostructure. Metabolomics analysis of the selected sponge followed by molecular docking studies, will be conducted to explore and predict the secondary metabolites that might provide its capability of inhibiting cancer. Materials and Methods: We prepared a chloroform extract (CE) and ethyl acetate extract (EE) of the Red Sea sponge *Phyllospongia lamellosa* synthesized silver nanoparticles. The prepared silver nanoparticles were characterized through UV–vis spectrophotometric, transmission electron microscopy (TEM), and Fourier-transform infrared spectroscopy (FTIR) analyses. Testing for their anti-cancer activities was performed against MCF-7, MDB-231, and MCF-10A cells. Anti-inflammatory activity against COX-1 and 2 was assessed. Furthermore, liquid chromatography–mass spectrometry (LC–MS)-based metabolomics analysis and molecular docking were also applied.

## 1. Introduction

Green methods of preparation of metallic nanoparticles (MNPs) are more preferred than chemical ones with many environmental consequences. These environment-friendly procedures include the use of mixed-valence polyoxometalates [1], polysaccharides [2], lesser irradiation [3], and biological methods [4,5].

Biological methods are among the most common methods in green preparation of MNPs [6]. In these methods, biological matrices are used as reducing agents to synthesize MNPs and, at the same time, as capping materials to keep them in a colloidal form [7]. Besides being eco-friendly, these methods are also highly economical. Moreover, they can add more value to the prepared MNPs due to the surface-adsorbed molecules [8].

Marine environments are vibrant ecosystems that contain many unique forms of life that may have unique chemical and biological properties with high potential therapeutic value. Sponges are among the most promising marine organisms with unprecedented complex chemistry and outstanding biological activities [9,10,11,12]. The potential use of marine organisms in the preparation of MNPs is underexplored in comparison with the terrestrial ones, particularly plants [5]. We believe that biological matrices derived from them have the capacity to synthesize MNPs by acting as reducing agents and can add extra therapeutic potential to the prepared MNPs through their unique chemical entities that can be adsorbed on the particles’ surface [13].

Accordingly, in this investigation, we explored the role of a crude organic extract derived from the marine sponge *Phyllospongia lamellosa* in the preparation of stable and bioactive silver nanoparticles (SNPs), consisting in the reduction of aqueous AgNO_3_. *P. lamellosa* has been previously reported to be an excellent source of bioactive compounds, particularly sesterterpenes, which have shown to have anti-cancer properties. SNPs synthesized by *P. lamellosa* extracts were then tested for their anticancer and anti-inflammatory activities.

## 2. Materials and Methods

### 2.1. Collection of Marine Sponge

The marine sponge *Phyllospongia lamellosa* (Esper, 1794) (3 kg) was collected in November 2020 from Hurghada along the Red Sea coast (27°15048″ north (N), 33°4903″ east (E)) at a depth of 7 m. A voucher sample (NIOF320/2016) was reserved at the Invertebrates Department, National Institute of Oceanography and Fisheries, Red Sea Branch, Hurghada, Egypt.

### 2.2. Preparation of Extracts

Sponge material was cut into small pieces (1 cm × 1 cm) and then subjected to ultrasonic-assisted extraction with ethanol, as mentioned in page S2 (Supplementary Material File 1).

### 2.3. Metabolomic Analysis

The recovered extracts were subjected to metabolic analysis using LC-HRESIMS according to Abdelmohsen et al., 2014 [14]. The details for the LC-HRESIMS method are described in Supplementary Material File 1, page S2.

### 2.4. Preparation of Silver Nanoparticles

The green synthesis of silver nanoparticles was carried out according to Alhadrami et al., 2021 [15] (Supplementary Material File 1, page S3).

### 2.5. Characterization of Silver Nanoparticles

#### 2.5.1. UV Spectroscopy

A color change of the solution visualized the synthesis of AgNPs. Then, the transformation of Ag^3+^ to Ag^0^ was monitored by periodic sampling of aliquots (1 mL) of the mixture and measuring the UV–vis spectra of the solutions by using a SPECTROstar nano absorbance plate reader (BMG LABTECH).

#### 2.5.2. X-ray Diffraction (XRD) Studies

The X-ray diffraction (XRD) patterns of the prepared green AgNPs were measured using a PANalytical X’pert PRO X-ray diffractometer (The Netherlands) with Cu Ka1 radiation under the operating voltage and tubing current of about 40 kV and 30 mA, respectively. The sample was drop-coated onto a glass substrate, and the X-ray diffraction patterns were recorded at 2θ from 10° to 80° with a scanning speed of 0.02°/min.

#### 2.5.3. Fourier-Transform Infrared Spectroscopy (FTIR)

The ATR-FTIR spectra of the silver nanoparticles were obtained using a Bruker vertex 80 V in a range of 4000–400 cm^−1^, with a resolution of 4 cm^−1^, according to Brock-Neely (1957).

#### 2.5.4. Transmission Electron Microscopy (TEM) Analysis

Transmission electron microscopy (TEM) was performed to examine the size and morphology of the synthesized silver nanoparticles. The sample preparation was performed by placing 2–4 µL of either silver or gold nanoparticle solution on carbon-coated copper grids. The thin film formed was air-dried under ambient conditions and observed using a Philips 10 Technai with an accelerating voltage of about 180 keV with a wavelength (λ) of 0.0251 Å.

#### 2.5.5. Scanning Electron Microscope (SEM)

Scanning electron microscopy was carried out using a field emission scanning electron microscope (FESEM), the Thermo Scientific™ Quanta FEG-250 (FEI, Eindhoven, The Netherlands)), at an acceleration voltage of 20 kV, along with energy dispersive X-ray analysis (EDAX) to detect the elemental composition.

### 2.6. Determination of the Antimicrobial Activity of Silver Nanoparticles

To measure the antibacterial activity of the nanoparticles, three Gram-negative bacteria (*Escherichia coli* ATCC 25955, *Proteus vulgaris*, and *Salmonella typhimurium*), one Gram-positive bacteria (*Staphylococcus aureus* NRRL B-767), one yeast (*Candida albicans* ATCC 10231) and one fungus (*Aspergillus niger*) were used as test organisms. In addition, antibacterial tests were performed as described in Supplementary Material file 1, page S3.

#### 2.6.1. Antiproliferative Assay

The antiproliferative activity of the prepared SNPs was tested as described in detail in Supplementary Material File 1, page S3.

#### 2.6.2. COX Inhibitory Assay

The in vitro inhibitory assays of the prepared SNPs against both COX-1 and COX-2 were carried out using fluorometric-based screening kits (Biovision, Milpitas, CA 95035 USA) according to the manufacturer’s protocol [16,17,18]. The procedure is described in detail in Supplementary Material File 1, page S3.

#### 2.6.3. In Silico Biological Activity Predictions

PASS [19] was employed to predict the most possible anticancer metabolites in the *P. lamellose*-derived extract and point a probable molecular target for them. The details for PASS were described in Supplementary Material File 1, page S4.

#### 2.6.4. Determination of the Potential Protein Targets of the Annotated Compounds

To determine the potential targets for the dereplicated compounds, we performed inverse docking against all proteins present in PDB [20]. The details are described in Supplementary Material File 1, page S4.

#### 2.6.5. Molecular Docking Experiments

Molecular docking was carried out using the Autodock Vina software [21,22]. The details are described in Supplementary Material File 1, page S4.

### 2.7. Statistical Analysis

All in vitro analyzes were performed in triplicate. Pooled data were given as mean ± standard error (SEM) of at least three independent experiments. The differences among various treatment groups were determined by ANOVA, followed by Dunnett’s test using the PASW^®^ Statistics, version 18 (Quarry Bay, Hong-Kong). A difference with a value of *p* < 0.001 was considered statistically significant compared with a vehicle-treated control group and depicted by an asterisk symbol. The IC_50_ values were detected using a nonlinear regression curve fitting analysis using the GraphPad Prism software, version 6 (La Jolla, CA, USA).

## 3. Results and Discussion

### 3.1. Chemical Profiling of P. lamellosa-Derived Extracts

**Figure 1:**
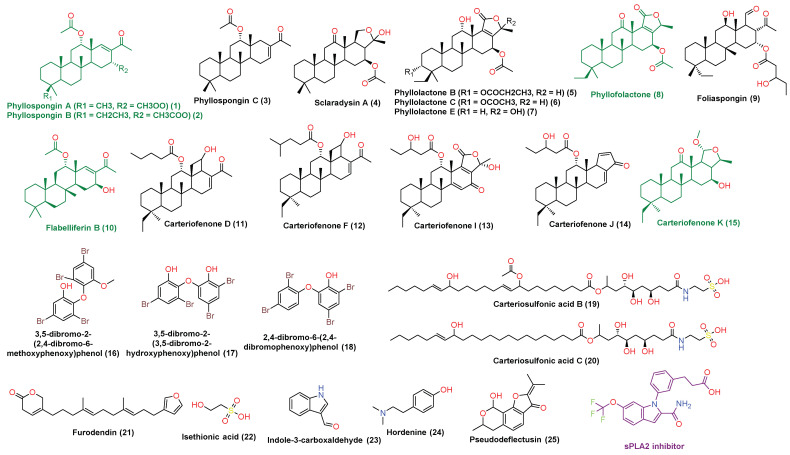
Chemical structures of the compounds 1–25 that were putatively identified in the *P. lamellosa*-derived extracts. Green-colored compounds were predicted to have anticancer activity and to inhibit sPLA2. The mauve-colored compound is the reported co-crystalized inhibitor of sPLA2.

Chemical characterization of *P. lamellosa*-derived extracts led to putative identification of 25 major compounds (Supplementary Material File 1 (Table S1), Figure 1). The compounds identified in CE (1–15) were found to belong to the terpenoids class of natural products (i.e., sesterterpenes and triterpenes), while in the EE, three brominated aromatic ethers (16–18), two sulfated fatty amides (19 and 20), and five miscellaneous compounds (21–25) were identified. All the identified compounds had been reported before as components of *P. lamellosa* or other *Phyllospongia* sp. [11,12,13]. Sesterterpenes and triterpenes (1–15) are very characteristic of *P. lamellosa* and have shown an interesting anticancer activity against several types of human cancer cell lines. In addition, they showed moderate anti-inflammatory and antiviral activities both in vitro and in vivo [11,12,13].

Carteriosulfonic acids B and C are two sulfated fatty amides identified in the EE (19 and 20, respectively). Both compounds have been reported to inhibit glycogen synthase kinase-3 beta (GSK-3β) in vitro, and hence, they are considered as very promising drug candidates for treatment of type 2 diabetes mellitus [23].

Loading such a crude extract that is rich in different interesting bioactive metabolites on MNPs, such as silver, has a great potential to maximize its bioactivity.

### 3.2. Biosynthesis of AgNPs Using Phyllospongia lamellosa Extracts

**Figure 2:**
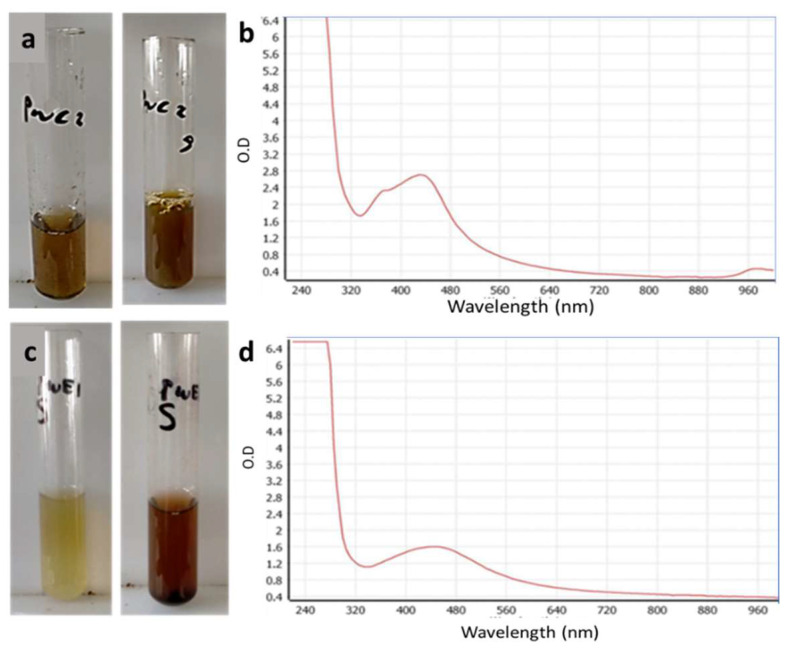
Color change due to formation of AgNPs by CE (**a**) and EE (**c**) and UV–vis spectra showing a clear plasmon band for AgNPs synthesized by PWC (**b**) and PWE (**d**).

The biosynthesis of silver nanoparticles was accompanied by a color change of the mixture. We observed a yellowish brown color in the case of CE and a reddish brown color in the case of EE (Figure 2a,c), both indicating the formation of nanoparticles. The reduction of the silver metal to nanometal in the presence of CE was monitored by observing the color change to brown due to the excitation of surface plasmon vibrations in the particles. Furthermore, the formation of AgNPs was also confirmed by UV–vis absorption spectroscopy. A characteristic maximum absorbance peak of AgNPs appeared at 440 nm because the EE extract has the ability to reduce the metal ions to their corresponding metal particles in the nanoscale range (Figure 2b).

### 3.3. Electron Microscopy

**Figure 3:**
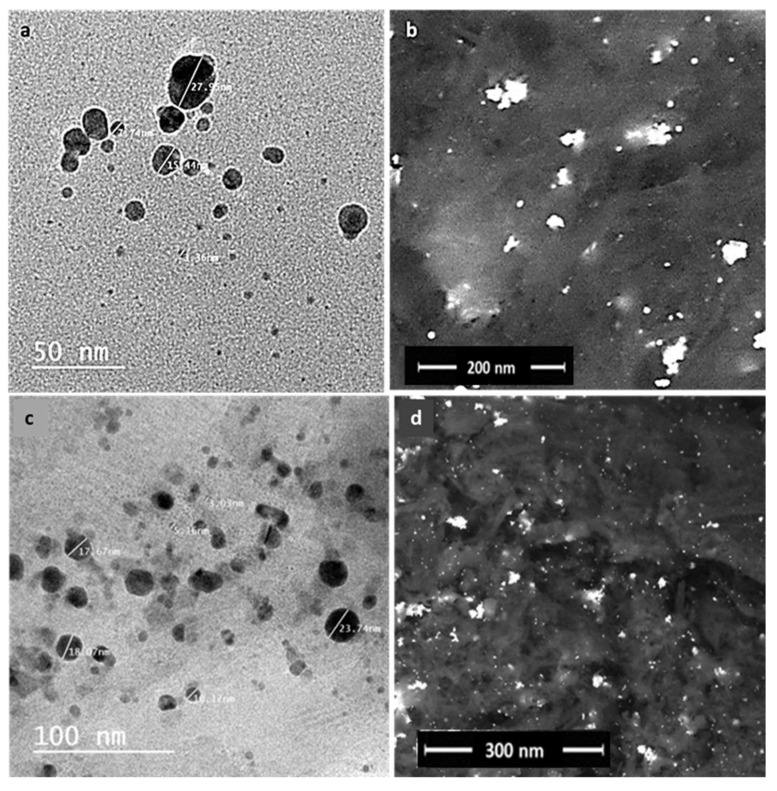
TEM micrographs of poly-dispersed-shaped AgNPs prepared with CE (**a**) and spherical-shaped AgNPs prepared with EE (**c**) and FESEM micrographs of the prepared AgNPs (**b**,**d**).

The particle size and morphology of the green synthesized AgNPs was measured via transmission electron microscopy (TEM) and field emission scanning electron microscopy (FESEM). The average particle size of the AgNPs in the case of CE was about ~2.47 ± 2 to 27.55 ± 2 nm and the AgNPs were spherical, while in the case of EE, it was about ∼5.16 ± 2 to 23 ± 74 nm and the shape was spherical-like (Figure 3a,b).

### 3.4. X-ray Powder Diffraction (XRD)

**Figure 4:**
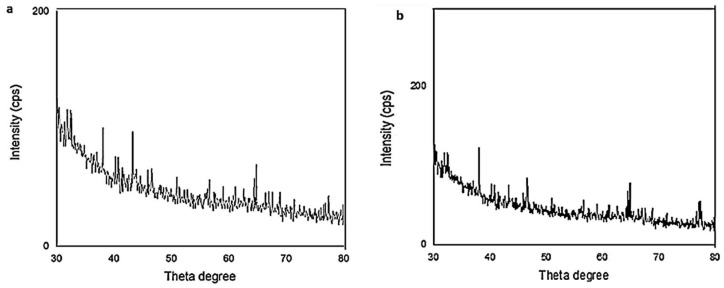
X-ray diffraction patterns of the silver nanoparticles prepared with CE (**a**) and EE (**b**).

X-ray powder diffraction is a highly versatile technique that provides chemical information for elemental analysis as well as for phase analysis. Therefore, X-ray diffraction (XRD) analysis was performed to study the structural properties of the green synthesized AgNPs. The XRD results for the CE-synthesized AgNPs showed that the most essential characteristic peaks of the Ag phase appeared at 38°, 44°, and 64°, corresponding to the crystallographic planes (111), (200), and (220) of silver, while the EE-synthesized silver nanoparticles showed the presence of characteristic peaks of silver at 38.04°, 43.32°, 64.35°, and 77.4°, corresponding to the crystallographic planes (111), (200), and (220) of silver (Figure 4).

### 3.5. Fourier-Transforms Infrared Spectroscopy Analysis (FTIR)

**Figure 5:**
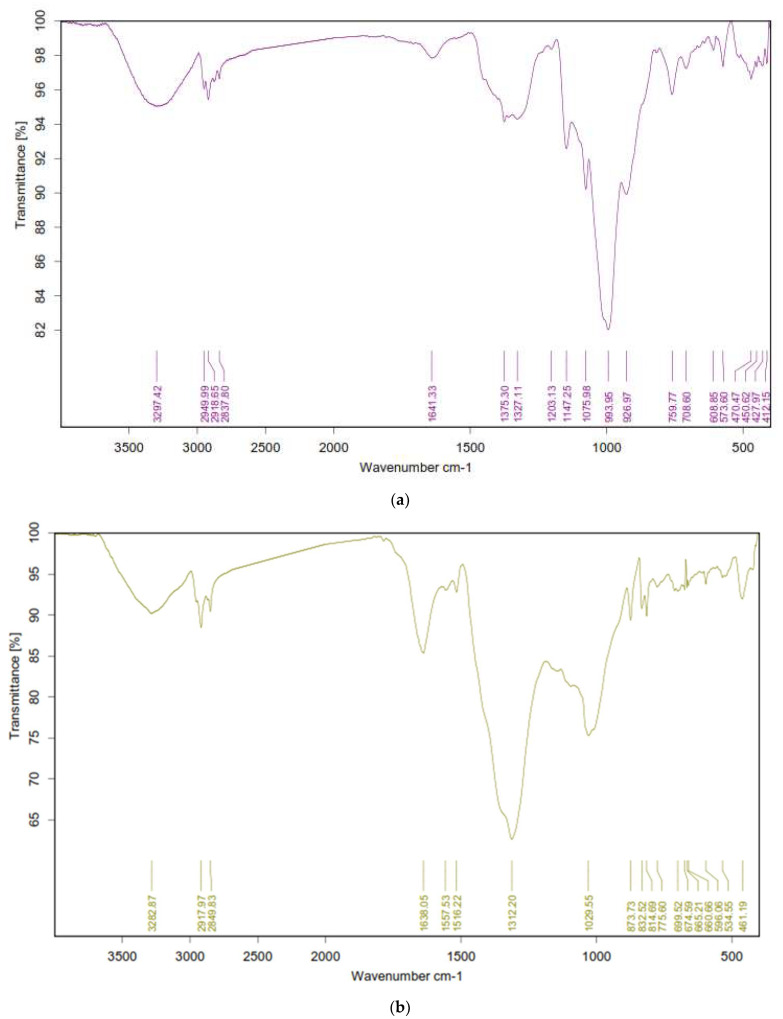
FTIR spectra of the green prepared AgNPs using CE (**a**) and EE extract (**b**).

The FTIR was performed to identify the functional groups responsible for synthesis and stabilization of silver nanoparticles. The FTIR spectral profile of the green AgNPs prepared with the CE extract showed that the hydroxyl group (O–H) stretching vibration appeared at 3297.42. In contrast, the peaks that appeared at 2949.99–2837.80 cm^−1^ referred to the alkane (C–H) stretching vibration. Moreover, the carbonyl (C=O) stretch vibration was displayed at 1641.33 cm^−1^. The C–O was measured at 1375.30–1327.11 cm^−1^. Additionally, the C–N appeared at 1203.13 and 1075.98 cm^−1^ (Figure 5). The presence of a band corresponding to C=C, C=O, C–O, C–N, OH, and CH suggested that they play a very important role in bioreduction of Ag metal ions and provide stability by acting as a capping agent to the formed AgNPs.

On the other hand, the results of the analysis of the EE extract in the presence of AgNPs showed that hydroxyl group (O–H) stretching vibration appeared at 3282.87 cm^−1^. In contrast, peaks that appeared at 2917.97 and 2849.83 cm^−1^ referred to the alkane (C–H) stretching vibration. Moreover, the carbonyl (C=O) stretch vibration was displayed at 1638.05 cm^−1^. The (C=N) stretch bands were observed at 1516.22 cm^−1^. The C–O was measured at 1312.20 and the band at 1218.83 cm^−1^ referred to C–N stretching. Additionally, the C–C appeared at 1029.55 cm^−1^. These functional groups, such as carboxyl, hydroxyl, and nitrogenous groups interact with the Ag metal ion and reduce it to Ag nanoparticles (Figure 5).

### 3.6. Target Prediction and Docking Analysis

Computer-based biological activity evaluation that depends on modern algorithms and methodologies (e.g., artificial intelligence and machine learning) has become widely incorporated into the drug discovery process. Such virtual and computer-aided procedures could be helpful in analysis of a natural crude extract [24,25,26].

Accordingly, we subjected the metabolites (1–25) identified in the *P. lamellosa*-derived extracts (i.e., CE and EE) to the neural networking-based prediction software PASS. This software has shown very good success rate in its predictions, particularly for predictions higher than Pa = 0.5 [27,28,29,30,31,32,33].

**Figure 6:**
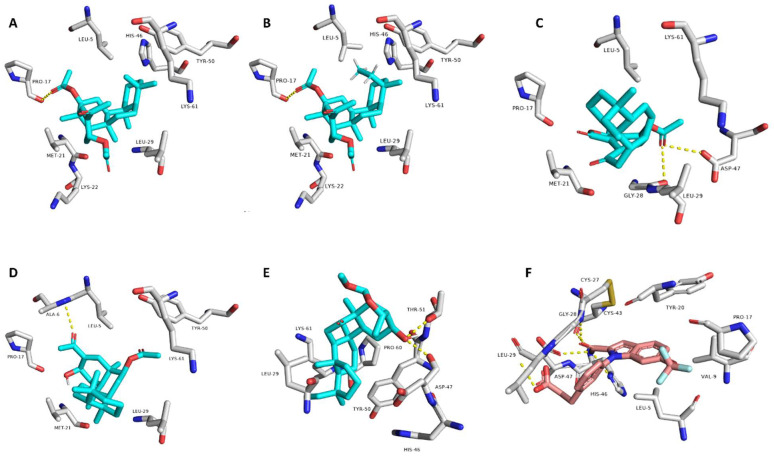
Predicted binding modes of compounds 1, 2, 8, 10, and 15 (**A**–**E**, respectively) inside sPLA2 (PDB code: 5OWC), alongside the co-crystalized inhibitor (**F**).

As depicted in Figure 6, only compounds 1, 2, 8, 10, and 15 that obtained Pa scores >0.5 and are highly likely to be active as anticancer compounds in vitro. Subsequently, we subjected all these compounds to an inverse docking-based screening to find out the most probable molecular target(s) that may mediate their predicted anticancer activity. Interestingly, compounds 1, 2, 8, 10, and 15, which were predicted to be active as anticancer agents, had also outstanding binding energy scores (<−6 kcal/mol) for binding to the secreted phospholipase A2 (sPLA2), comparable with that of its co-crystalized inhibitor. In addition, they were able to establish H-bonds with ALA-6, PRO-17, THR-51 and also with ASP-47 and LEU-29 like the co-crystalized ligand. Moreover, they showed a number of hydrophobic interactions with LEU-5, MET-21, HIS-46, TYR-50, and LYS-6 (Figure 6).

sPLA2 has been reported to have a crucial role in the growth and development of different types of human cancers (e.g., breast, prostate, liver, and skin), and identified to be a promising target to control these types of tumors [22,24,25,26,27,28,29,30,31,32,33,34].

**Figure 7:**
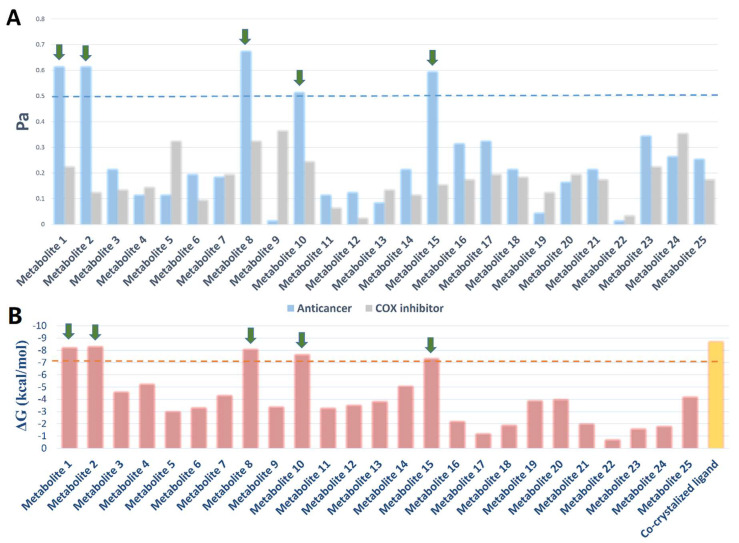
PASS prediction scores of the compounds 1–25 as anticancer agents and COX inhibitors (**A**). Pa > 0.5 indicates high probability of them being active in vitro. Binding energy scores of compounds 1–25 inside sPLA2 (**B**).

It is worth noting that the compounds 1–15 have also shown to be COX inhibitors according to PASS prediction; however, their scoring was poor (Pa < 0.5) (Figure 7).

### 3.7. In Vitro Biological Activity of SNPs

#### In Vitro Anticancer Activity

According to our virtual screening analysis, the *P. lamellosa*-derived extract showed probable anticancer potential. Hence, our prepared SNPs, loaded with this extract, have high potential to be more effective than the free ones.

Accordingly, we tested all the papered SNPs against two human breast cancer cell lines, estrogen-positive (MCF-7) and multidrug resistant (MDA-MB-231), as well as against normal breast cells (MCF-10a), to evaluate the toxicity of these SNPs.

**Figure 8:**
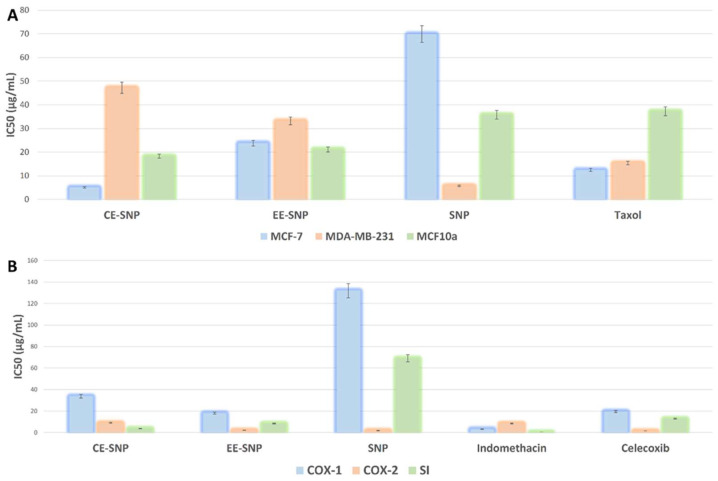
In vitro growth inhibitory activities of the prepared SNPs against estrogen-positive and triple negative breast cancer cell lines alongside a normal breast cell line (**A**), and their inhibitory activities against both COX-1 and COX-2 (**B**). IC_50_ values were expressed as µg/mL ± SE. CE-SNP—SNPs loaded with chloroform extract; EE-SNP—SNPs loaded with ethyl acetate extract; SNP—free SNPs; SI—selectivity index.

As shown in Figure 8A, both SNPs loaded with CE and EE showed very good inhibitory activity against MCF-7 (IC_50_ = 5.1 ± 0.24 and 23.8 ± 2.47 µg/mL, respectively). In addition, they were significantly more active than the free ones (IC_50_ = 69.9 ± 3.24 µg/mL). Interestingly, CE-SNP were more potent than the reference drug, taxol (IC_50_ = 12.47 ± 1.25 µg/mL), and this indicates the potential of sesterterpenes and triterpenes that were identified in CE as potent anticancer agents.

Regarding the multidrug-resistant strain (MDA-MB-231), the free SNPs were far more active (IC_50_ = 5.78 ± 0.27 µg/mL) than the extracts-loaded ones (IC_50_ = 47.2 ± 3.19 and 33.2 ± 3.6 µg/mL in the cases of CE and EE, respectively). The toxicity of all prepared SNPs toward normal breast cells was moderate (IC_50_ ranged from 18.3 ± 2.85 to 35.8 ± 3.2 µg/mL) and comparable with that of taxol (Figure 8A).

These results indicated that the chemical constituents present in *P. lamellosa*-derived extracts, particularly in CE, had a significant anticancer effect against estrogen-sensitive breast cancer cells and enhanced the activity of the SNPs. In contrast, these chemical constituents were inactive against multidrug-resistant breast cancer cells. Moreover, they even hindered the superior activity of the free SNPs.

### 3.8. In Vitro COX Inhibitory Activity

To evaluate the in vitro anti-inflammatory activity of our prepared MNPs, we tested them for their COX inhibitory activity. All MNPs showed moderate to weak inhibitory activity against both COX-1 and COX-2, except for the activities of free SNPs and SNPs loaded with sponge extracts (i.e., CE and EE) against COX-2, which were very low. Additionally, they showed very good selectivity indices (SI) that were better than those of the used reference drugs (Figure 8B).

These interesting activities against COX-2 were possibly due to the action of the SNPs themselves, and the sponge extract had likely only a weak or even no effect.

## 4. Conclusions

In the present study, the LC–HRESIMS-assisted chemical profiling of *P. lamellosa* extract revealed that this marine sponge is rich in terpenoids-based natural products. Utilizing this extract in the green synthesis of MNPs led to the preparation of bioactive SNPs that exerted interesting anticancer properties with superior activity against estrogen-sensitive human breast cancer with low to moderate cellular toxicity. Virtual screening of the identified metabolites in the sponge extract highlighted a number of sesterterpenes and triterpenes to be the potentially active chemical entities. It also suggested that these active metabolites may exert their anticancer activities via targeting sPLA2. Further isolation and purification of the active components from the sponge crude extract together with the in vivo studies are in progress to find the applicability of such a formulation as an anti-cancer therapeutic agent.

## Supplementary Materials

The following are available online at https://www.mdpi.com/article/10.3390/antibiotics10101155/s1, Supplementary Material File 1.
