# Supplementary material for: Anti-Cancer and Anti-Inflammatory Potential of the Green Synthesized Silver Nanoparticles of the Red Sea Sponge Phyllospongia lamellosa Supported by Metabolomics Analysis and Docking Study
Source: Antibiotics (Basel). 2021 Sep 24;10(10):1155. doi: 10.3390/antibiotics10101155 (PMC8532725; doi:10.3390/antibiotics10101155)
Supplement: Supplementary file 1 [file antibiotics-10-01155-s001.zip › antibiotics-1370783-supplementary.pdf]

Supplementary Material File 1

Article

Anti-Cancer and Anti-Inflammatory Potential of the Green Synthesized Silver Nanoparticles of the Red Sea Sponge *Phyllospongia lamellosa* Supported by Metabolomics Analysis and Docking Study

Areej A. Al-Khalaf [1,*,†], Hossam M. Hassan [2,3,*,†], Aisha M Alrajhi [1], Rania Ali El Hadi Mohamed [1] and Wael N. Hozzein [4,5]

[1] Department of Biology, College of Science, Princess Nourah bint Abdulrahman University, Riyadh 11671, Saudi Arabia; amo.alrajhi@gmail.com (A.M.A.); r.ali2012@yahoo.com (R.A.E.H.M.)
[2] Department of Pharmacognosy, Faculty of Pharmacy, Nahda University, Beni-Suef 62513, Egypt
[3] Department of Pharmacognosy, Faculty of Pharmacy, Beni-Suef University, Beni-Suef 62514, Egypt
[4] Bioproducts Research Chair, Zoology Department, College of Science, King Saud University, Riyadh 11671, Saudi Arabia; hozzein29@yahoo.com
[5] Botany and Microbiology Department, Faculty of Science, Beni-Suef University, Beni-Suef 62514, Egypt
* Correspondence; aaalkhalaf@pnu.edu.sa (A.A.A.-K.); hossam.mokhtar@nub.edu.eg (H.M.H.)
† These authors contributed equally to this work.

1. Preparation of extracts

Sponge material was cut into small pieces and then subjected to ultrasonic-assisted extraction with ethanol as mentioned (3 × 500 mL). The resulting alcoholic extract was concentrated using a rotary evaporator (IKA®, Staufen, Germany). The obtained concentrated extract was suspended in distilled water and fractionated with chloroform (4 × 200 mL) and ethyl acetate (4 × 200 mL) to give chloroform and ethyl acetate extracts (CE and EE, respectively). All fractions were separately concentrated under reduced pressure and kept at 4˚C.

2. Metabolomic Analysis Procedure

The prepared crude-extract was dissolved in methanol to reach a concentration of 1mg/mL-for-mass-spectrometry-analysis. An-Acquity-Ultra-Performance-Liquid-Chromatography-system attached to a-Synapt-G2-HDMS-quadrupole-time-of-flight hybrid mass spectrometer (Waters,-Milford,-MA, USA) was utilized. Positive and negative ESI-ionization-modes-were-employed-to obtain-the- connected high-resolution-mass-spectrometry, with a-spray-voltage-at-4.5-kV, the-capillary temperature-at-320°C, and mass-range-from-*m*/*z*-150–1500. The MS-dataset-was-processed-and-data-were obtained utilizing MZmine-2.20-based-on the accepted parameters. Mass-ion-peaks were identified and-accompanied-by-chromatogram-builder-and-chromatogram-deconvolution. The local minimum search algorithm was addressed, and isotopes were also analyzed via the isotopic peaks of grouper. Missing peaks were displayed using the gap-filling peak finder. An adduct-search-along-with-a complex-search-was carried out. The-processed-dataset was later exposed to-molecular-formula-prediction-and-peak-identification. The positive and negative ionization mode datasets from the respective extract were dereplicated against-the-DNP-(Dictionary-of-Natural-Products) databases.

Table S1. Dereplicated-metabolites-from-LC-HRESIMS analysis of *P. lamellosa* crude-extracts.

No.	Compound Name	Original source	MF	RT (min.)	Accurate Mass
		**Chloroform Extract**			
1	Phyllospongin A	*P. lamellosa*	$C_{29}H_{44}O_5$	7.8	472.3185
2	Phyllospongin B	*P. lamellosa*	$C_{30}H_{46}O_5$	7.6	486.3345
3	Phyllospongin C	*P. lamellosa*	$C_{27}H_{42}O_3$	8.01	414.3138
4	Scl aradysin A	*P. lamellosa*	$C_{28}H_{44}O_5$	8.58	460.3185
5	Phyllolactone B	*P. lamellosa*	$C_{32}H_{48}O_7$	8.56	544.3403
6	Phyllolactone C	*P. lamellosa*	$C_{31}H_{46}O_7$	8.59	530.3244
7	Phyllolactone E	*P. lamellosa*	$C_{29}H_{44}O_6$	8.71	488.3141
8	Phyllofolactone	*P. lamellosa*	$C_{29}H_{42}O_5$	9.51	470.3035
9	Foliaspongin	*P. lamellosa*	$C_{23}H_{52}O_6$	9.98	532.3760
10	Flabelliferin B	*P. lamellosa*	$C_{27}H_{42}O_4$	7.82	430.3081
11	Carteriofenone D	*Phyllospongia* sp.	$C_{32}H_{50}O_4$	9.61	498.3705
12	Carteriofenone F	*Phyllospongia* sp.	$C_{33}H_{52}O_4$	9.65	512.3861
13	Carteriofenone I	*Phyllospongia* sp.	$C_{32}H_{46}O_7$	9.11	542.3240
14	Carteriofenone J	*P. lamellosa*	$C_{32}H_{48}O_4$	9.35	496.351
15	Carteriofenone K	*P. lamellosa*	$C_{28}H_{46}O_4$	6.5731	446.3394
		**Ethyl acetate Extract**			
16	3,5-dibromo-2-(2,4-dibromo-6-methoxyphenoxy)phenol	*Phyllospongia* sp.	$C_{13}H_8Br_4O_3$	4.54	527.7205
17	3,5-dibromo-2-(3,5-dibromo-2-hydroxyphenoxy)phenol	*Phyllospongia* sp.	$C_{12}H_6Br_4O_3$	5.68	513.7055
18	2,4-dibromo-6-(2,4-dibromophenoxy)phenol	*Phyllospongia* sp.	$C_{12}H_6Br_4O_2$	4.54	497.7104
19	Carteriosulfonic acid B	*Phyllospongia* sp.	$C_{38}H_{69}NO_{12}S$	6.90	763.4545
20	Carteriosulfonic acid C	*Phyllospongia* sp.	$C_{34}H_{65}NO_{10}S$	7.27	679.4326
21	Furodendin	*Phyllospongia* sp.	$C_{22}H_{30}O_3$	4.73	342.2191

22	Isethionic acid	*Phyllospongia* sp.	C2H6O4S	2.11	125.9986
23	Indole-3-carboxaldehyde	*Phyllospongia* sp.	C9H7NO	2.67	145.0527
24	Hordenine	*Phyllospongia* sp.	C10H15NO	2.72	165.1151
25	Pseudodeflectusin	*Phyllospongia* sp.	C15H16O4	4.23	260.1044

3. Preparation of Silver Nanoparticles

The green synthesis of silver nanoparticles was carried out according to ***Abdelhafez* et al., 2020, in**. Briefly, .0.005g crude extracts was dissolved in 1 mL of dimethyl sulfoxide. An amount of 0.5 mL of both the extracts solution were mixed with 25 mL of 1 mM $AgNO_3$.The mixture was stirred at 70 ˚C for 5 h in the dark until the change of the mixture color to yellowish brown indicated the reduction of AgNO3.

4. Determination of the antimicrobial activity of silver nanoparticles

To measure the antibacterial activity of the nanoparticles, three Gram-negative bacteria (Escherichia coli ATCC 25955, Proteus vulgaris, and Salmonella typhimurium), one Gram-positive bacteria (*Staphylococcus aureus NRRL B-767*), one yeast (Candida albicans ATCC 10231) and one fungus (*Aspergillus niger*) were used as test organisms and antibacterial tests were performed. The tests were performed in 96-well flat polystyrene plates. An amount of 10µl of test extracts (final concentration of 50 µg/ml) were added to 80 µl of lysogeny broth (LB broth) followed by the addition of 10 µl of bacterial culture suspension (log phase), then the plates were incubated overnight at 37°C. After incubation, the positive antibacterial effect of the tested compound was observed as clearance in the wells, while in compounds that did not have an effect on the bacteria, the growth media appeared opaque in the wells. The absorbance was measured after about 20 h at OD600 in a Spectrostar Nano Microplate Reader (BMG LABTECH GmbH, Allmendgrun, Germany).

5. Anticancer activity

Cell lines (breast (MCF-7), liver (HepG2), and colorectal (CACO2) cancer cell lines) were maintained in earth RPMI 1640 medium containing 10% heat-inactivated fetal bovine serum (FBS) and were grown at 37 °C and 5% CO2. Cell lines were counted and seeded on 96-well cell culture plates (2 × 104 cells/well in the case of MCF-7 and HepG2 and 6 × 104 cells/well in the case of CACO2 cells). Cells were stimulated with the indicated concentrations of each compound overnight in triplicate. Cell viability was assessed either by 3-(4,5-dimethylthiazol-2-yl)-2,5-diphenyltetrazolium bromide (MTT) assay 8. To normalize cell viability values, each plate included a triplicate of untreated cells considered as 100% viability, and a triplicate of cells treated with a cytotoxic mixture (200 ng/mL TNF, 200 ng/mL CD95L, 200 ng/mL TRAIL, 5 µg/mL CHX, 1% (w/v) sodium azide 20%) considered as 0% viability. All other viability values were normalized according to the averages of these triplicates and analyzed by the Graph Pad Prism 5 software (La Jolla, CA, USA).

6. COX inhibitory assay

These assays are based on the detection of the florescence produced by prostaglandin G2 (i.e., the intermediate product produced by the COX1 and 2 enzymes). The enzyme solutions were prepared by adding 110 mL of ddH_2O to the lyophilized enzymes in the kit. The diluted COX cofactor was prepared by mixing the COX assay buffer (398 mL) and COX Cofactor (2 mL). An amount of 5 mL of arachidonic acid were added to 5 mL of NaOH and then diluted by 90 mL of ddH_2O to produce dilute arachidonic acid/NaOH solution. Subsequently, All of these prepared solutions were mixed together to produce the reaction mixture (80 mL). Different concentrations of the test MNPs were added to the previous solution. The reaction mixtures were then incubated at 25 ˚C for 10 min. The produced florescence (Ex/Em = 535/587 nm) was measured by Tecan Spark microplate reader (Tecan Instruments, Inc., New York, USA). These assays of test MNPs, blank, and reference inhibitors were carried out in triplicate. IC_{50} values were calculated by GraphPad

software (version 7.0), where the percentage inhibitions were plotted versus the log concentrations. Selectivity index (SI) was calculated by dividing the IC_{50} calculated for COX-2 by the IC_{50} calculated for COX-2.

7. In Silico Biological Activity Predictions

PASS was employed for the prediction of the most possible anticancer metabolites in *P. lamellosa*-derived extract, and to suggest a probable molecular target for them. This-software was capable of -predicting->4000-types of pharmacological-and-toxicological-activities, including their mechanism-of-action, with approximately-85% acceptable-precision, depending-on-the submitted compound-structures that were-subsequently screened-by applying the-structure-activity relationship-database (SARBase). The prediction-results-were given as probability-scores (probably active "Pa" or probably inactive "Pi"). These-calculated probability-scores were determined by-linking the-structure and-functional groups-features in the tested-molecules that matched or-mismatched the specific activities recorded-in the software-associated-database. The higher-the Pa-values, the more-acceptable it was-for the-compound to-present the suggested pharmacological-activity on a scale of 0–1. Pa values higher-than 0.5 meant a high-experimental chance-of the suggested-pharmacological activity.

8. Determination of the Potential Protein Targets of the annotated Compounds

Potential Protein targets for the identified compounds were proposed by subjecting all of these compounds to inverse docking against all proteins hosted in the Protein Data Bank (PDB; https://www.rcsb.org/, accessed on: 13 July 2021). The idTarget platform (http://idtarget.rcas.sinica.edu.tw/, accessed on: 13 July 2021) was used for this task. This structure-based screening software applies a unique docking approach called divide-and-conquer docking that adaptively builds small overlapping grids to make the searching space on the protein surfaces more constrained, and hence, it can run a huge number of accurate docking experiments in a much reduced time [20]. The retrieved results were obtained as a list of binding affinity scores arranged from the heist negative value to the lowest one. We set a binding affinity score of -7 kcal/mol as a cut-off value to select the best targets for each identified compound in CE.

9. Molecular Docking Experiments

Molecular docking was carried out-utilizing-Autodock-Vina [21]-software. The crystal structures of human secreted phospholipase A2 (sPLA2) (PDB codes: 5OWC) were retrieved from-the protein-databank-website-(https://www.rcsb.org/, accessed on: 13 July 2021) [22], and later developed using-Autodock-tools software.-The binding site of the co-crystalized ligand was selected for-the-docking-experiments. The co-ordinates of the grid box were: x = -12.87; y = 16.3; z = 68.64. The size of the grid box was set to be 10 Å. Exhaustiveness was set to be 24.10, and poses were generated for each docking experiment. Top-scoring binding-poses with-Root Mean-square-Deviation (RMSD) values-less than 2 Å were then-selected and visualized-using Pymol-software [21].

References

20. Wang, J.C., Chu, P.Y., Chen, C.M., Lin, J.H. idTarget: A web server for identifying protein targets of small chemical molecules with robust scoring functions and a divide-and-conquer docking approach. *Nucleic Acids Res*, **2012**, *40*, W393–W399.
21. Seeliger, D., de Groot, B.L. Ligand docking and binding site analysis with PyMOL and Autodock/Vina. *J Comput Aided Mol Des*, **2010**, *24*, 417–422.
22. Knerr, L., Giordanetto, F., Nordberg, P., Pettersen, D., Selmi, N., Beisel, H.G., de la Motte, H., Olsson, T., Perkins, T.D.J., Herslöf, M., et al. Discovery of a series of indole-2 carboxamides as selective secreted phospholipase A2 type X (sPLA2-X) inhibitors. *ACS Med Chem Lett*, **2018**, *9*, 594–599.
